# Traditional Chinese Mind and Body Exercises for Promoting Balance Ability of Old Adults: A Systematic Review and Meta-Analysis

**DOI:** 10.1155/2016/7137362

**Published:** 2016-11-21

**Authors:** Shihui Chen, Yanjie Zhang, Yong Tai Wang, Xiao Lei Liu

**Affiliations:** ^1^Department of Kinesiology, Texas A&M University Texarkana, TX, USA; ^2^Department of Physical Education, Chinese University of HK, Shenzhen, China; ^3^College of Nursing and Health Science, University of Texas at Tyler, Tyler, TX, USA; ^4^Department of Traditional Sports, Beijing Sports University, Beijing, China

## Abstract

The purpose of this study was to provide a quantitative evaluation of the effectiveness of traditional Chinese mind and body exercises in promoting balance ability for old adults. The eligible studies were extensively searched from electronic databases (Medline, CINAHL, SportDicus, and Web of Science) until 10 May 2016. Reference lists of relevant publications were screened for future hits. The trials used randomized controlled approaches to compare the effects of traditional Chinese mind and body exercise (TCMBE) on balance ability of old adults that were included. The synthesized results of Berg Balance Scale (BBS), Timed Up and Go Test (TUG), and static balance with 95% confidence intervals were counted under a random-effects model. Ten studies were selected based on the inclusion criteria, and a total of 1,798 participants were involved in this review. The results of the meta-analysis showed that TCMBE had no significant improvement on BBS and TUG, but the BBS and TUG could be obviously improved by prolonging the intervention time. In addition, the results showed that TCMBE could significantly improve the static balance compared to control group. In conclusion, old adults who practiced TCMBE with the time not less than 150 minutes per week for more than 15 weeks could promote the balance ability.

## 1. Introduction

Today, the issue of aging is a major public concern worldwide. According to recent population census [1], Chinese aged 60 years or older were more than 220 million, about 15% of the total population by the end of 2014. The numbers of old population will continue to grow and reach 437 million by 2051. The similar situation exists in the United States; for example, the numbers of population aged over 65 will reach 85 million by 2050 and will be doubled compared with the number in 2010 [2]. Many aging related studies showed health issues which increase prevalence of chronic diseases, complex medical conditions, and even loss of their independent functions [3–5]. Issue of aging brings serious economic burden to the society, of which the elderly's health and medical related costs are the major expenditures for them [4, 6]. Participating in exercises and physical activities may have beneficial effects on the aging related chronic diseases and health conditions of elderly people. There is good evidence that appropriate physical exercise can effectively prevent the onset of noncommunicable diseases and can improve the health condition and fitness of elderly people [7]. Previous studies found that exercise therapy as nonpharmacological intervention may have beneficial effects on functional recovery, such as balance, strength, walking gait correction, and fall prevention [8–11].

Traditional Chinese mind and body exercises (TCMBE) include, for example, Tai Chi, Qigong, Baduanjin, or Yijinjing, all developed by ancient Chinese people. TCMBE as an effective, low-cost, and safety exercise approach is widely accepted in elderly people in China and other Asian countries [12–14]. During recent years, many studies reported that motor movements of elderly people were improved by participating in Tai Chi, Qigong, and Baduanjin [10, 15, 16]. Besides, Verhagen et al. [17] and Voukelatos et al. [18] reported that practicing Tai Chi can effectively reduce the falls of elderly people. Wenneberg et al. [19] provided evidence that persons who practice Qigong exercise could significantly improve their balance control abilities. The decline of balance function is the main reason of causing fall for the elderly [13]. Therefore, the TCMBE program may be an appropriate exercise to improve elderly people's balance ability and reducing the risk of falls.

This systematic review and meta-analysis aimed to summarize the current research results of randomized controlled trials on the effectiveness of TCMBE on balance and the risk to fall in the healthy elderly. This study analyzed the effectiveness of TCMBE intervention program compared with control group (non-TCMBE) on balance ability in elderly populations.

## 2. Materials and Methods

### 2.1. Search Strategy

The Preferred Reporting Items for Systematic Reviews and Meta-Analysis guideline (PRISMA) was followed throughout this review and research processes. Four databases of literature had been used as data sources for this study from inception until 10 May, 2016 (Medline: 1946-, SportDicus: 1978-, Cinahl: 1992-, and Web of Science: 1900-). The two groups of terms were combined for the systematical search as follows: (1) “traditional Chinese exercise” OR “tai chi” OR “tai chi chuan” OR “taiji” OR “taijiquan” OR “qi gong” OR “chi kung” OR “Baduanjin” OR “Yijinjing”; AND (2) “balance” OR “balance control” OR “fall” OR “falls” OR “slip”. In addition to the database search, a manual search from the reference lists of identified articles and relevant articles was also applied. Further, additional studies were also found from many experts who were in the field of traditional Chinese exercise.

### 2.2. Inclusion Criteria

The included studies should meet the following criteria: (1) type of studies: the current research only applied randomized controlled trials; (2) participants: the study participants were healthy humans with age over 60 years; (3) interventions: the subjects of study must be related to a treatment group that only using traditional Chinese exercise and a control group involved in no TCMBE exercises or other treatment (e.g., wellness education, and resistance training). There must be different interventions between treatment group and control group (either passive or positive); (4) outcome measures: the outcomes were related to balance performance, for instance, Berg Balance Score (BBS) and Timed Up and Go Test (TUG); (5) providing adequate information for calculating effect size; (6) the fact that traditional Chinese exercise intervention period was no less than two months (8 weeks).

### 2.3. Exclusion Criteria

The studies were excluded if (1) there was no randomized assignment to study groups; (2) the trial used the Chinese exercise plus additional treatments (e.g., Tai Chi and stretch training in treatment group); (3) the presence of animal models or unhealthy participants (e.g., diabetic and stroke participants); (4) there is a lack of sufficient information to calculate the effect size; (5) they were questionnaire, case study, abstract, or reviews; and (6) the studies were published in non-English journals.

### 2.4. Selection of Studies

The literature searches were conducted by two authors. The two reviewers independently screened the potential articles by reading the titles and abstracts and then full-text articles according to the eligible criteria. Disagreements between two reviewers were resolved in discussion. If necessary, the third reviewer was consulted to reach a consensus.

### 2.5. Data Extraction

The following information from each article was extracted: (1) author and year of publication; (2) characteristic of the participants: sample size, sex, and age; (3) study design; (4) interventions; and (5) standardized mean, standard deviation, or raw data for effect size calculation. There were no disparities of data abstraction.

### 2.6. Quality Assessment

Two reviewers independently assessed the quality of study using the Jadad scale [20]. This scale has been widely applied in assessing methodological quality in exercise field [21, 22]. The scale includes three items: randomization (0–2 points), blinding (0–2 points), and withdrawals and dropouts (0-1 point). The range scores of Jadad were 0–5. A score higher than 3 points could be considered as high-quality studies.

### 2.7. Statistical Analysis

The Comprehensive Meta-Analysis software was used for meta-analysis. The *Q*-test and the I2-coefficient were used to examine heterogeneity between studies. If there is a statistic significant *Q*, it indicates differences of study heterogeneity. And the I2 statistic was used to measure the effect of heterogeneity with low (25%), moderate (50%), and high (75%) respectively [23]. The fixed-effects model was conducted to pool ESs with 95% confidence interval (CI), if the heterogeneity was not found. Otherwise, the random-effects model was adopted. Eventually, the publication bias was examined through using the Egger regression asymmetry test and Begg's funnel plot.

## 3. Results

### 3.1. Search Results

**Figure 1:**
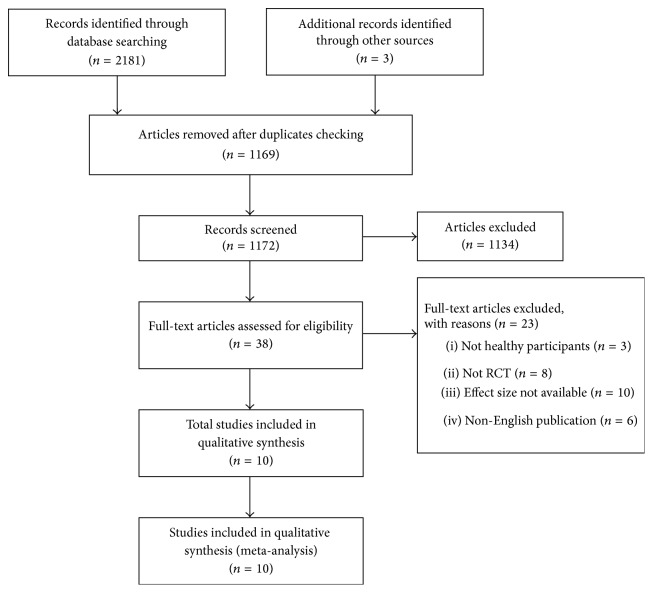
Flow diagram for selection of studies.

Figure 1 shows the flow diagram of the study selection process. A total of 2,184 articles were found in Medline, CINAHL, SportDicus, and Web of Science. After removing duplications, 1169 articles were eliminated, and 1134 articles were further excluded after screening the titles and abstracts. The remaining studies (*n* = 38) were reviewed for eligibility through reading the full-texts. Finally, 10 studies (12 trials, two studies included two eligible trails) were included in the present review [12, 16, 24–31].

### 3.2. Study Characteristics

The main characteristics of eligibility studies are summarized in Table 1. All included articles were published between 2003 and 2015, and they aimed to measure the effectiveness of traditional Chinese mind and body exercise on balance or fall prevention among healthy old adults. A total of 1,798 participants were involved in 10 studies. The sample size ranged from 40 to 702 participants. Three types of interventions were used: eight studies (10 trials) used Tai Chi, one study used Tai Chi + Qigong [27], and one study used Baduanjin [16].

### 3.3. Risk of Bias within Studies

The main risks of bias of the included studies are summarized in Table 2. It indicated that six trials had a high bias risk and six trials had a low bias risk based on the Jadad scale recommendation. All of the studies used the randomized allocation, but three studies reported using appropriate randomization methods, and the rest studies used appropriate double blinding or double blinding. In addition, most trials from these studies explicitly stated the number of withdrawals or the reasons for dropout. Both passive and active control group are included in the selected studies, and the ordinary intervention (not TCMBE) or other treatment (e.g., wellness education, and resistance training) was used for the control group. Eight trials used regular daily exercise [24–26, 28–31] and four trials used the wellness education, regular daily care exercises, and regular physiotherapy intervention [12, 16, 27, 31]. The detailed conditions and activities arranged for the control group are listed in Table 1.

### 3.4. Synthesis of Results and Berg Balance Scale

**Figure 2:**
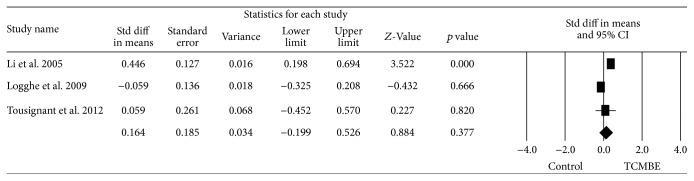
The effect of TCMBE versus control on BBS for the elderly.

In this meta-analysis the main outcome measures were Berg Balance Scale (BBS); Timed Up and Go Test (TUG); and static balance.  Figure 2 shows the forest plot of the meta-analysis. Three trials stated the BBS for old people, and a random-effects model was used due to the high heterogeneity: *Q* = 7.68, *p* = 0.02, and *I* ^2^ = 73.98%. The pooled results indicated that there was no significant improvement on BBS in favor of elderly people practicing the traditional Chinese exercise (TCMBE): ES = 0.164, 95% CI (−0.199, 0.526), *z* = 0.88, and *p* = 0.38.

### 3.5. Timed Up and Go Test

**Figure 3:**
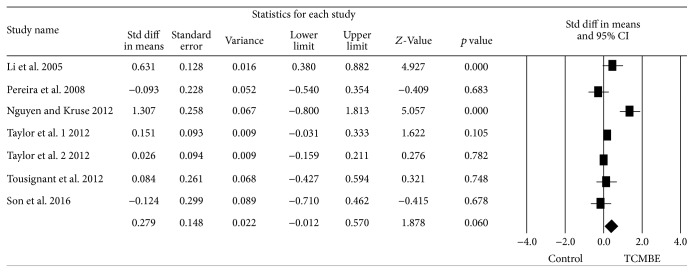
The effect of TCMBE versus control on TUG for the elderly.

There were seven trials in which the TUG was conducted in this meta-analysis. According to estimating the synthesis results, significant heterogeneity was found among the eligible seven trials, *Q* = 36.27, *p* < 0.01, and *I* ^2^ = 83.46%, and then a random-effects model was conducted to calculate the effect size.  Figure 3 shows the forest plot of the analysis. The pooled results did not indicate any effect of TCMBE on TUG for the elderly: ES = 0.28, 95% CI (−0.01, 0.57), *z* = 1.88, and *p* = 0.06.

### 3.6. Static Balance

**Figure 4:**
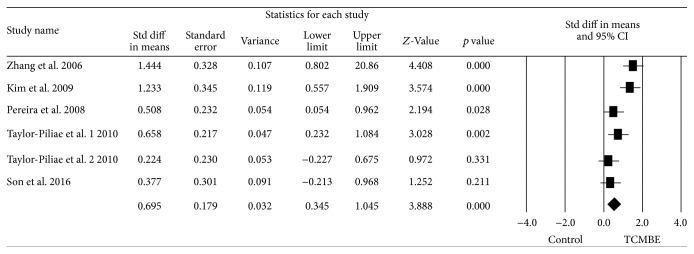
The effect of TCMBE versus control on static balance for the elderly.

Static balance was reported in six trials in this meta-analysis. After calculating the pooled results in Figure 4, it gives a significant heterogeneity: *Q* = 13.34, *p* < 0.01, and *I* ^2^ = 65.51%, so the random-effects model was suited for this analysis. It demonstrated that the static balance ability of the elderly in TCMBE group was significantly improved compared to the control group: ES = 0.70, 95% CI (0.03, 0.35), *z* = 3.89, and *p* < 0.01.

### 3.7. Publication Bias

**Figure 5:**
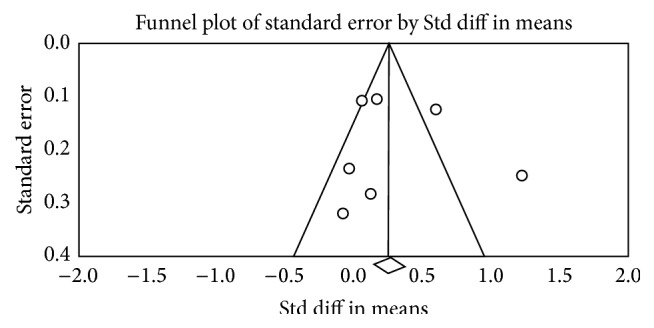
Funnel plot (TUG).

**Figure 6:**
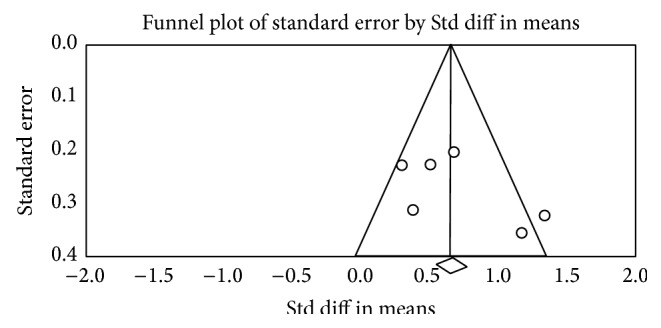
Funnel plot (static balance).

In aspect of TUG and static balance, the nonsignificant results of Egger's test stated no publication bias with *p* = 0.31 and *p* = 0.06, respectively. But the two funnel plots were not symmetrical (Figures 5 and 6). The possible reason for unsymmetrical plot may be because of the heterogeneity among the included studies rather than publication bias.

### 3.8. Subgroup Analysis

**Figure 7:**
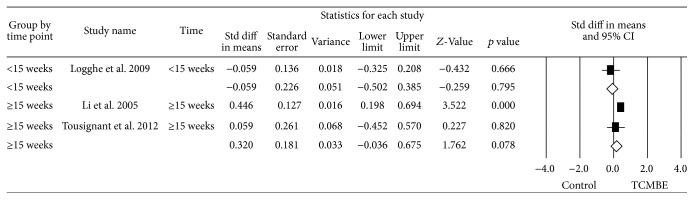
Subgroup meta-analysis: the effect of TCMBE versus control on BBS for elderly TUG.

**Figure 8 fig8:**
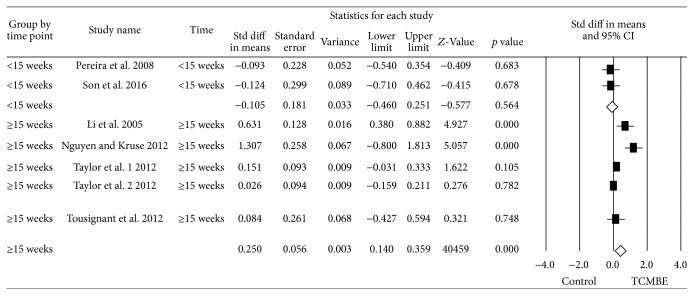
Subgroup meta-analysis: the effect of TCMBE versus control on TUG for the elderly.

**Figure 9:**
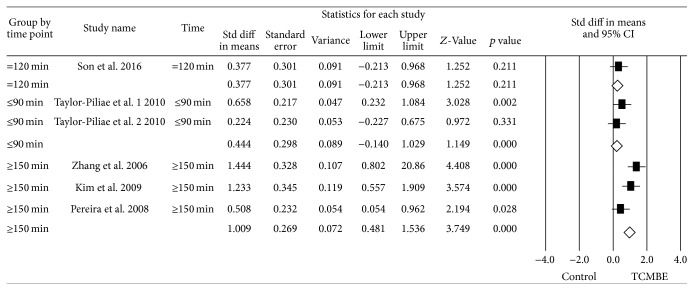
Subgroup meta-analysis: the effect of TCMBE versus control on static balance for the elderly.

Due to the significant heterogeneity in BBS, TUG, and static balance, three subgroup analyses were performed to compare the different intervention period (less than 15 weeks and more than 15 weeks) and different exercise time per week (≤90 minutes, around 120 minutes, and ≥180 minutes). To perform the subgroup analysis, the random-effects model was used. The forest plots of the subgroup analysis were shown in Figures 7 [Fig fig8]–9.

Firstly, there was no difference in TCMBE group on BBS compared with control group in intervention period (*Q* = 1.70, *p* = 0.20). However, the potential result was longer period (≥15 weeks) and TCMBE had an effect on BBS (ES = 0.32, *p* = 0.001). Secondly, it indicated TCMBE group had no effect on TUG compared with control group based on the different duration time (*Q* = 2.11, *p* = 0.15). There also was a trend that long period (≥15 weeks) TCMBE play a positive effect on TUG. Finally, no group difference in exercise time per week (*Q* = 2.53, *p* = 0.28) was found. But the evidenced result was that if the participants took a longer time (≥150 minutes per week) to participate in TCMBE, they could improve their static balance power.

## 4. Discussion

This systematic review and meta-analysis was conducted to assess the effectiveness of TCMBE intervention compared with control group (non-TCMBE activity) on balance ability and risk to fall for old adults. We found that (1) there was no effect on BBS by using the TCMBE, based on the synthesis ESs of 3 randomized controlled trials; (2) TCMBE intervention did not significantly improve old adults' TUG though displaying the pooled ESs of seven randomized controlled trials; and (3) there was a significant improvement on static balance power, in particular, if the subjects participate in the TCMBE program for more than 150 minutes per week. These findings seem to be different from the previous meta-analysis [8, 14, 32], in which Tai Chi intervention significantly improved the BBS and TUG compared with control group. The current study includes more eligible trials using the TCMBE interventions and its characteristics are homogenous compared to previous meta-analysis, such that if one study has three different groups, we use the TCMBE group to compare with the other two groups, respectively. Besides, the previous review included the participants with diseases, such as stoke, dementia, and Parkinson's. Our study used the healthy population without any disease. In addition, the current review firstly evidenced the effect of TCMBE on static balance ability for old adults though meta-analysis.

Because of the high heterogeneity in meta-analysis, we conducted subgroup meta-analysis to investigate the reasons of heterogeneity. The intervention time (≥15 weeks) of participants participating in the TCMBE showed significantly effect on BBS and TUG for old adults (*p*_BBS_ = 0.001 and *p*_TUG_ = 0.02, resp.). There was significantly improved static balance for old adults if the time they participated in the TCMBE was not less than 150 minutes per week (*p* < 0.01). Therefore, more senior people are encouraged to take part in the TCMBE program with long practicing time every week in order to achieve the benefits of the TCMBE exercise.

This meta-analysis study has few limitations that may influence the findings; for example, there are 50 percent of trials that are classified as high bias risk based on the Jadad scale. The present review only consists of English publications. The varied TCMBE sessions per week and the different measurement parameters were used in original studies. Moreover, in original studies some participants dropped out the experiment at the end of the trial; the number of participants at the postintervention was used to assess the ESs. This systematic review and meta-analysis did not evaluate the long-term beneficial effects of TCMBE on balance for the elderly after the end of intervention. It is expected that more studies are needed to confirm the findings in the future.

## 5. Conclusion

In summary, the time old adults participated in the TCMBE program more than 150 minutes per week for more than 15 weeks can promote their balance ability. Therefore, the traditional Chinese mind and body exercise training program can be introduced to the public as an alternative rehabilitation treatment for old adults to promote their balance ability.

## Figures and Tables

**Table 1:** Characteristics of eligible studies in current meta-analysis.

Author and year	Subjects	Duration	Design	Intervention		Outcome	Key findings
				Control	Experiment		
Li et al. 2005	*n* = 256 Age: 70–92 Gender: 77M, 179F	24 w	RCT	Stretching 60 minutes, 3 times per week	Tai Chi 60 minutes, 3 times per week	BBS TUG	The score of BBS and TUG was improved in the Tai Chi group compared to the stretching group

Zhang et al. 2006	*n* = 49 Age: ≥60 Gender: 25M, 24F	8 w	RCT	Continue their current level of physical activity	Tai Chi 60 minutes, 7 times per week	One-leg balance	TCC training group significantly improved in one-leg balance compared to daily activities

Pereira et al. 2008	*n* = 70 Age: 60–82 Gender: 77F	12 w	RCT	Doing their daily activities	Tai Chi 50 minutes, 3 times per week	Unipodal position test	TC had an increase in unipodal position compared to control group

Kim et al. 2009	*n* = 40 Age: 65–87 Gender: 20M, 20F	12 w	RCT	Wellness education 60 minutes, per week	Tai Chi 60 minutes, 3 times per week	Center of pressure	TC had a greater COP compared to wellness education

Logghe et al. 2009	*n* = 269 Age: ≥70 Gender: 78M, 191F	13 w	RCT	Usual care	Tai Chi + Chi Kung 60 minutes, twice per week	BBS	No difference

Taylor-Piliae et al. 2010 Trial 1	*n* = 132 Age: 60–84 Gender: 40M, 92F	24 w	RCT	Attend-control 90 minutes, once per week	Tai Chi 45 minutes, twice per week	Single-leg stance	Tai Chi had greater improvements compared to control group

Taylor-Piliae et al. 2010 Trial 2	*n* = 132 Age: 60–84 Gender: 40M, 92F	24 w	RCT	Western exercise 45 minutes, 3 times per week	Tai Chi 45 minutes, twice per week	Single-leg stance	Tai Chi had greater improvements compared to WE

Nguyen and Kruse 2012	*n* = 96 Age: 60–79 Gender: 48M, 48F	24 w	RCT	Daily activities	Tai Chi 60 minutes, twice per week	TUG	TUG in TC group is significantly improved compared to daily activities

Taylor et al. 2012 Trial 1	*n* = 684 Age: ≥65 Gender: 182M, 502F	20 w	RCT	Low-level exercise 60 minutes, once per week	Tai Chi 60 minutes, once per week	TUG	No difference

Taylor et al. 2012 Trial 2	*n* = 684 Age: ≥65 Gender: 182M, 502F	20 w	RCT	Low-level exercise 60 minutes, once per week	Tai Chi 60 minutes, twice per week	TUG	No difference

Tousignant et al. 2012	*n* = 152 Age: ≥65 Gender: 41M, 111F	15 w	RCT	Physiotherapy intervention	Baduanjin 60 minutes, twice per week	BBS TUG	No difference

Son et al. 2016	*n* = 50 Age: 65–83 Gender: 50F	12 w	RCT	Otago exercise 60 minutes, twice per week	Tai Chi 60 minutes, twice per week	TUG OLS	Otago group exhibited a greater improvement in TUG compared to TC group; TC group showed greater improvement in OLS compared to Otago group

Note: TC: Tai Chi; TUG: Timed Up and Go Test; BBS: Berg Balance Score; OLS: One-leg-standing.

**Table 2:** Quality assessment of included studies.

Author and year	Randomization	Double blinding	Withdrawals	Appropriate randomization	Appropriate double blinding	Total
Li et al. 2005	1	0	1	1	0	3
Zhang et al. 2006	1	0	1	0	0	2
Pereira et al. 2008	1	0	1	0	0	2
Kim et al. 2009	1	0	0	0	0	1
Logghe et al. 2009	1	1	1	0	1	4
Taylor-Piliae et al. 2010 Trial 1	1	0	1	0	0	2
Taylor-Piliae et al. 2010 Trial 2	1	0	1	0	0	2
Nguyen and Kruse 2012	1	0	1	0	0	2
Taylor et al. 2012 Trial 1	1	0	1	1	1	4
Taylor et al. 2012 Trial 2	1	0	1	1	1	4
Tousignant et al. 2012	1	0	1	0	1	3
Son et al.2016	1	0	1	1	1	4
